# Differences in Expression, Content, and Activity of 11**β**-HSD1 in Adipose Tissue between Obese Men and Women

**DOI:** 10.5402/2012/787201

**Published:** 2012-12-09

**Authors:** A. Torres, G. Iñiguez, M. Ferrario, V. Mericq

**Affiliations:** ^1^Institute of Maternal and Child Research, Faculty of Medicine, University of Chile, Casilla 226-3, 8360160 Santiago, Chile; ^2^Department of Surgery, Clínica Las Condes, Santiago, Chile

## Abstract

Cortisol production in adipose tissue is regulated by 11**β**-HSD1. *Objective*. To determine whether there are differences in gene expression, enzyme activity, and protein content of the 11**β**-HSD1 enzyme in VAT (visceral adipose tissue) and SAT (subcutaneous adipose tissue) from obese compared to nonobese adults. *Methods*. VAT and SAT samples were obtained from 32 obese subjects (BMI > 30 Kg/m^2^) who underwent bariatric surgery and 15 samples from controls submitted to elective surgery. Fasting serum glucose, insulin, and lipids were measured. The expression of 11**β**-HSD1 was determined by RT-PCR, the enzyme activity by thin-layer chromatography, and the protein content by Western blot. *Results*. Obese patients had higher cholesterol, insulin, and HOMA-IR compared to nonobese. There were no differences in VAT or SAT expression of 11**β**-HSD1 between obese and nonobese patients. However, we found lower 11**β**-HSD1 activity and protein content in VAT, in obese women versus nonobese women (*P* < 0.05). BMI and 11**β**-HSD1 enzyme activity and protein content in VAT correlated inversely in women. *Conclusions*. Regulation of 11**β**-HSD1 activity in VAT from obese subjects appears to be gender specific, suggesting the existence of a possible protective mechanism modulating this enzyme activity leading to a decrease in the production of cortisol in this tissue.

## 1. Introduction 

Obesity is a major health problem around the world, and its prevalence has increased rapidly. It is defined as the presence of an excessive amount of body fat, which is associated with morbidity and mortality [1–3]. During the last 10 years, evidence has accumulated that strongly argues for an etiological role of 11*β*-hydroxysteroid dehydrogenase type 1 (11*β*-HSD1) in obesity and metabolic syndrome (MS) [4]. 

The 11*β*-HSD1 (EC 1.1.1.146) is a low-affinity nicotinamide adenine dinucleotide NADP(H)-dependent enzyme belonging to the superfamily of short-chain dehydrogenases/reductases, located within the endoplasmic reticulum, which catalyzes the interconversion of biologically inactive glucocorticoid (cortisone) to active glucocorticoid (cortisol) by oxidoreductase activity [5]. This enzyme is expressed in liver, muscle, central nervous system, placenta, lung, bone, kidney, gonads, and adipose tissue [6–8]. Physiological actions of cortisol include the regulation of protein synthesis and metabolism, as well as carbohydrates, lipids, and nucleic acids [9].

Glucocorticoid excess promotes visceral obesity and cardiovascular disease [10, 11]. Cortisol has an effect on the metabolism of carbohydrates elevating glycemia and acting as an antagonist of insulin. Similar features are found in the metabolic syndrome in the absence of high levels of systemic cortisol.

Transgenic mice that overexpress 11*β*-HSD1 selectively in adipose tissue develop visceral obesity, dyslipidemia, insulin resistance diabetes, and hypertension, whereas homozygous 11*β*-HSD1 knock-out mice are protected from obesity and the development of MS [12]. It has been hypothesized that a deregulation of the 11*β*-HSD1 and autocrine generation of cortisol in adipocytes could be involved in the pathogenesis of central obesity. 

Currently, there is no clear consensus regarding which fat depots, that is, visceral or subcutaneous adipose tissue, have the highest expression/activity of 11*β*-HSD1. In addition, controversy exists as to whether these differences are observed in obese versus control subjects and the impact in their metabolic profile. 

Thus our aim was to study the gene expression, enzyme activity, and protein content of 11*β*-HSD1 enzyme in a depot-specific manner in the visceral adipose tissue (VAT) and subcutaneous adipose tissue (SAT) of obese adults of both genders who underwent bariatric surgery compared to the nonobese group and the relationship to their fasting samples of metabolic parameters.

## 2. Materials and Methods

Samples of adipose tissue were collected from obese adult subjects. Inclusion criteria were a BMI > 30 kg/m^2^ and an indication for elective laparoscopic bariatric surgery. Nonobese, control subjects were chosen from adult nonobese patients, with a BMI < 30 kg/m^2^, who underwent elective surgeries for cholecystectomy, nephrectomy, or gynecologic reasons. Exclusion criteria for both groups of patients were age under 18 years or older than 65 years, hepatic disorders, diabetes, emergency surgery, and any disorder affecting energy metabolism, such as metabolic syndrome (MS), Cushing's or Addison's disease, polycystic ovarian syndrome (PCOS), and thyroid-related disorders. 

In all subjects, previous to the surgery, a fasting blood sample was drawn. Anthropometric information (age, weight, and height) were obtained from the clinical data of each subject. 

During the procedure, 5 g of visceral adipose tissue (VAT) and 5 g of subcutaneous adipose tissue (SAT) were obtained. The samples were obtained from the Obesity Surgery Center of Clínica Las Condes and San Borja-Arriarán Clinical Hospital in Santiago, Chile. The study protocol was approved by the Institutional Review Board of Clínica Las Condes and all patients gave written informed consent at recruitment.

### 2.1. Adipose Tissue Processing

During surgery a sample of adipose tissue from subcutaneous and visceral compartments was obtained. Both samples were cleaned, divided immediately, and frozen in liquid nitrogen and kept at –80°C for total RNA extraction and protein analysis.

### 2.2. Serum Biochemical Assays

Serum insulin was determined by IRMA (DIAsource, Belgium), with an intra- and interassay coefficients of variation (CV) of 2.1 and 4.5%, respectively. Glucose was measured by the glucose oxidase method from Roche Diagnostics (Mannheim, Germany), with an intra-assay and interassay CV of 2.5%. Serum total cholesterol, triglycerides, and HDL and LDL cholesterol were quantified by the Reflotron System of Diagnosis (Roche Diagnostics). Triglycerides were enzymatically measured using a spectrophotometric method. The intra- and interassay CV were 1.9 and 3.7%, respectively. Insulin sensitivity (IS) was estimated from fasting insulin (I0) and glucose levels using the homeostasis model (HOMA-IR) [13].

### 2.3. Expression Assays

#### 2.3.1. Total RNA Isolation and Quantitation

Total RNA was extracted from approximately 100 mg of VAT and SAT using TRIzol reagent (Invitrogen Corp., CA, USA) according to the manufacturer instructions. Adipose tissue pieces were homogenized in 1 mL of TRIzol supplemented with glycogen (0.25 mg/mL, Amersham Life Sciences) using mechanical homogenizer (Kontes Glass Company, Vineland, NJ, USA) the phases were separated by centrifugation (12000 rpm for 20 min at 4°C) after addition of chloroform. For RNA precipitation, isopropanol was added to the supernatant and centrifuged at 12000 rpm by 15 min at 4°C. The pellet was washed in 500 uL ethanol 75%. RNA was resuspended in diethylpyrocarbonate-treated water. RNA integrity was assessed by electrophoresis on 2% (w/v) agarose gels, and quantity was determined spectrophotometrically in a NanoDrop ND-1000 (NanoDrop Technologies, Wilmington, DE, USA). Complementary DNA was synthesized from 2 *μ*g of total RNA digested previously by DNAse I (Fermentas, USA) using random primers (Invitrogen) and 200 U reverse transcriptase RevertAid H Minus M-MuLV (Fermentas), following the manufacturer's instructions.

2 *μ*L cDNA was used for 11*β*-HSD1 amplification adjusted to a total volume of 25 *μ*L by adding PCR buffer containing 3 mmol/LMgCl_2_, 0.63 U Taq DNA polymerase (Invitrogen), a nucleotide mix, and 0.4 *μ*mol/L of each of two specific primers (upstream: 5′′-AGGAAAGCTCATGGGAGGACTAG-3′′ and downstream: 5′′-ATGGTGAATATCATCATGAAAAAGATTC-3′′). The reaction was performed in Thermocycler PT-100 (MJ Research Inc., Watertown, MA, USA) using conditions that were previously standardized: denaturing at 94°C for 1 min; annealing at 55°C for 1 min; extension at 72°C for 1 min 30 seg and repeating them for 31 cycles. 

As an internal control, 18S rRNA cDNA was amplified in each sample at the same conditions described above except for 1.5 mmol/L MgCl_2_ and repeated for 18 cycles. To determine that the amplification of all genes was within a linear range, we evaluated the linearity of amplification of the corresponding transcripts in adipose tissue and we subsequently selected the number of cycles. Amplicons of (139 bp, for 11*β*-HSD1 and 191 bp for 18S rRNA) were visualized on 2% agarose gel using GelRed Nucleic Acid Stain (Biotium). Semi-quantification of PCR products was performed by image analysis (KODAK EDAS 290 Electrophoresis Documentation and Analysis System, Kodak 1D Image Analysis Software). Results are expressed as the ratio between mRNA studied gene/18S rRNA (AU = arbitrary units).

### 2.4. Enzymatic Assays

**Protein Extraction**

VAT and SAT tissues were homogenized in ice-cold 0.1 M PBS pH 7.5 supplemented with antiproteases (Complete, Mini, EDTA-free Protease Inhibitor Cocktail Tablets, Roche Applied Science). The tissue homogenate was then centrifuged at 12000 rpm for 30 min at 4°C and the resulting supernatant was collected and assayed for protein concentration using the BCA protein assay kit (Pierce, Rockford, IL, USA) with BSA as a standard.

#### 2.4.1. Enzymatic Activity of 11*β*-HSD1

The enzymatic assay was previously described by Mericq et al. [14], modified to match the type of tissue under study. Reaction conditions were established using different concentrations of cofactor, unlabelled cortisone, and time of incubation. Linearity of the enzymatic reaction was also evaluated. 

Briefly, 200 *μ*g of protein extract were incubated in 0.5 mL phosphate buffer (0.1 mol/L pH 7.6) in the presence of 25000 c.p.m. of [3]-cortisone (Amersham, UK), 0.05 *μ*M cortisone, and 400 *μ*M NADPH (Sigma Chemicals). The reaction was initiated with the cofactor addition for 24 h at 37°C in a shaking water bath. Aliquots were extracted into 7 volumes of dichloromethane, and steroids were separated by high-performance thin-layer chromatography (HPTLC, Merck), using methanol-chloroform (8 : 92) as a mobile phase. The bands containing the labeled cortisone and cortisol were identified by UV light of the cold carriers, cut, and counted in a scintillation counter (Tracor Analytic Delta 300). The rates of cortisone to cortisol were calculated from the specific activity of the labeled cortisol and the radioactivity of cortisol (picograms) formed per mg protein per hour.

#### 2.4.2. Western Blot Analysis

Equal amounts (12.5 *μ*g) of adipose proteins were resolved by electrophoresis using 14% SDS-polyacrylamide gels and then transferred to nitrocellulose membranes (Bio Rad Laboratories, Hercules, CA, USA). The membranes were blocked with 5% BSA in TBS-T (20 mmol/L Tris pH 7.2, 137 mmol/L NaCl, 0.1% (v/v) Tween-20) for 1 h at room temperature. Blots were probed with antibodies against 11*β*-HSD1 (Santa Cruz Biotechnology, Santa Cruz, CA, USA) and *β*-actin (Sigma-Aldrich, St. Louis, MO, USA). After extensive washing, bands were detected with the appropriate horseradish peroxidase-conjugated secondary antibodies (Rockland Immunochemical Research, Gilbertsville, PA, USA) followed by enhanced chemiluminescence (ECL plus Western Blotting Detection System; Amersham Biosciences, Little Chalfont, UK). The images were acquired and evaluated using the UltraQuant Image Acquisition and Analysis Software (Ultralum Inc., Claremont, CA, USA), normalized relative to *β*-actin and expressed as arbitrary units (AU).

#### 2.4.3. Statistical Analyses

Results are shown as mean ± SEM. According to the data distribution between a *t*-test or a Mann-Whitney test was used to compare groups and paired *t*-test or Wilcoxon test for intragroup comparisons. Correlations studies were performed using the Pearson or Spearman test according to data distribution. Statistics were performed using SPSS v11.5.

## 3. Results

Thirty two obese (16 M, 16 W) and 15 nonobese subjects (7 M, 8 W) were included in this study. Anthropometric and biochemical characteristics of obese and nonobese subjects separated by gender are shown in Table 1. As expected, weight and BMI were higher in obese subjects, compared to their counterpart (*P* < 0.05).

No differences were found between obese subjects and nonobese in the concentrations of glycemia and triglycerides. Nevertheless, a higher concentration of total cholesterol and insulin and a higher HOMA-IR in the obese compared to nonobese subjects (*P* < 0.05) was detected. When separated by gender, insulin and HOMA-IR were higher in obese males compared to nonobese males, and in women only higher triglycerides in obese compared to nonobese were observed (Table 1).

### 3.1. Adipose Tissue Expression of 11*β*-HSD1

No differences were found in gene expression of the enzyme 11*β*-HSD1 in VAT and SAT within the obese and within the nonobese subjects. In addition, no differences were found in VAT gene expression of the enzyme 11*β*-HSD1 neither between the obese and nonobese group nor in the SAT from the obese compared to their counterparts.

**Figure 1:**
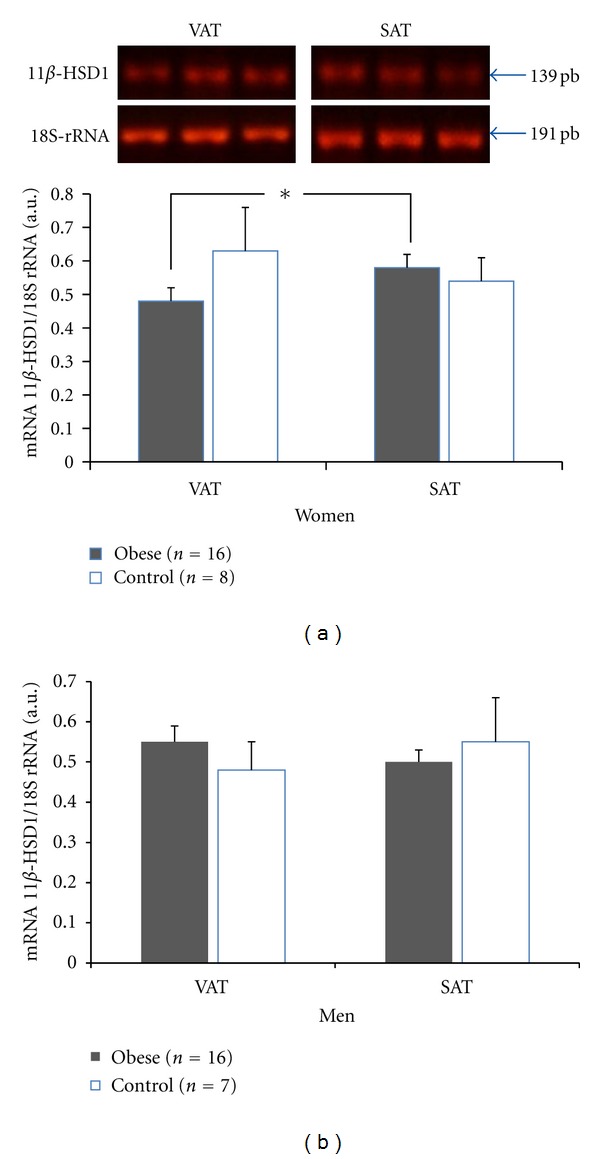
RT-PCR of 11*β*-HSD1. mRNA levels in visceral adipose tissue (VAT) and subcutaneous adipose tissue (SAT) separated by gender. (a) Women, (b) men. The data are expressed as mean ± SEM.

Data analyzed by gender showed no difference in gene expression of the enzyme 11*β*-HSD1 in VAT of obese women (0.48 ± 0.04 AU) compared to nonobese women (0.63 ± 0.13 AU) (Figure 1(a)) or of obese men (0.55 ± 0.04 AU) compared to nonobese men (0.48 ± 0.07 UA) (Figure 1(b)). The same was observed for SAT in men (0.50 ± 0.03 versus 0.55 ± 0.11 AU, resp.) and women (0.58 ± 0.04 versus 0.54 ± 0.07 AU). However, 11*β*-HSD1 mRNA expression in VAT of obese women was lower than that in SAT (*P* < 0.05) (Figure 1(a)). This difference in expression was not observed in men. 

### 3.2. Adipose Tissue Protein Levels of 11*β*-HSD1

**Figure 2:**
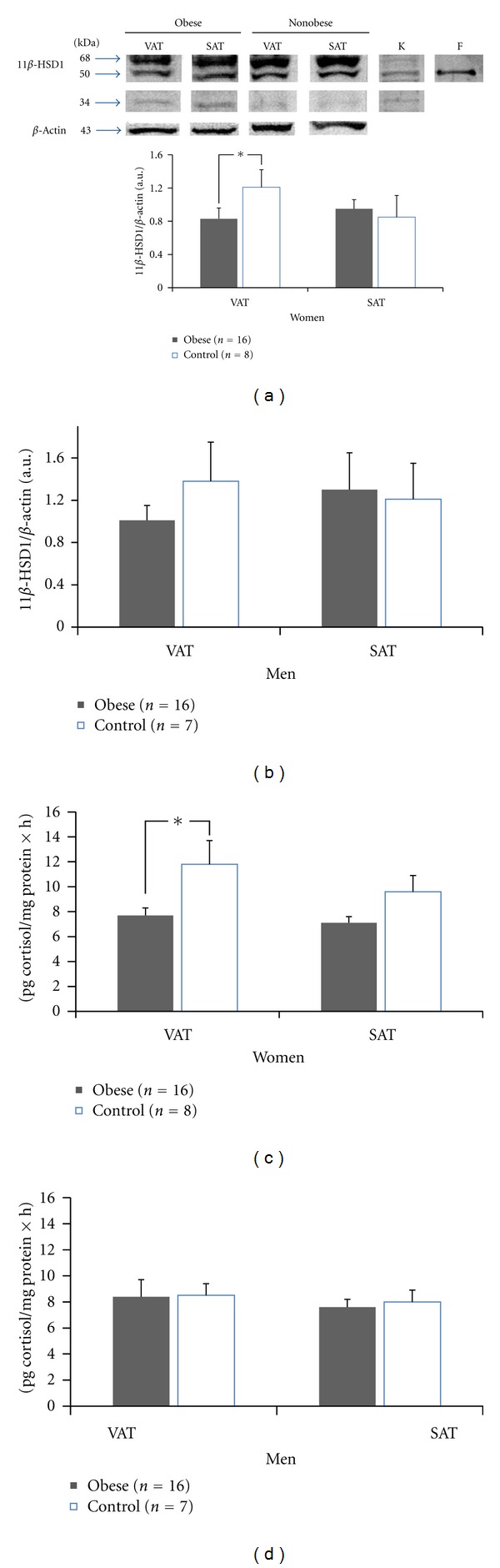
Protein levels and activity of 11*β*-HSD1. Protein levels and enzyme activity in visceral adipose tissue (VAT) and subcutaneous adipose tissue (SAT) separated by gender. (a, c) Women, (b, d) men, respectively. The data are expressed as mean ± SEM. K: kidney F: human skin fibroblast.

A representative image of Western blot analysis in adipose tissue, using a specific antibody, is shown in Figure 2 for the 11*β*-HSD1 with a molecular weight of 34 kDa (expected band) and two additional bands of 50 and 68 kDa. 

The protein content of the 34 kDa isoform of 11*β*-HSD1 of VAT was not different in obese and nonobese subjects (0.35 ± 0.08 versus 0.41 ± 0.17 AU resp., *P* = ns), as well as in SAT (0.14 ± 0.04 versus 0.06 ± 0.05). The additional band of 50 kDa has been previously described in human adipose tissue as an isoform of 11*β*-HSD1 [15, 16]. In our study, this 50 kDa form was more abundant than the 34 kDa isoform. This band was also observed in human skin fibroblast and kidney (Figure 2).

Similar to what we observed with the expression of mRNA of 11*β*-HSD1, no differences were found in the 34 kDa protein content of the enzyme 11*β*-HSD1 in VAT and SAT between the obese and nonobese subjects. Nevertheless, we found a lower 50 kDa protein content of 11*β*-HSD1 in VAT of obese compared to nonobese women (0.83 ± 0.13 versus 1.21 ± 0.21 AU, *P < 0.05) (Figure 2(a)). In women, this difference was not observed in SAT. These findings were not observed in men (Figure 2(b)). 

An additional band of 68 kDa corresponding to the dimeric form of 11*β*-HSD1, previously described in this tissue [15, 16], in VAT and SAT did not differ, between obese and nonobese men or women (data not shown). 

### 3.3. Adipose Tissue Activity of 11*β*-HSD1

The activity of the enzyme 11*β*-HSD1 in VAT of obese was lower than the activity of the enzyme in the nonobese subjects (data not shown). No differences were found in the enzyme activity in SAT of obese compared to nonobese subjects.

Figure 2(c) shows the activity of the enzyme 11*β*-HSD1 in VAT in obese women, which was lower than the activity of the enzyme in the nonobese women (7.7 ± 0.6 versus 11.8 ± 1.9 pg cortisol/mg of protein × h *P < 0.05). This difference was not observed in SAT of obese (7.1 ± 0.5 pg cortisol/mg protein × h) compared to nonobese women (9.6 ± 1.3 pg cortisol/mg protein × h) (Figure 2(c)). In men, no differences were observed in the activity of the enzyme 11*β*-HSD1 in both VAT and SAT of obese compared to nonobese men (Figure 2(d)).

**Figure 3:**
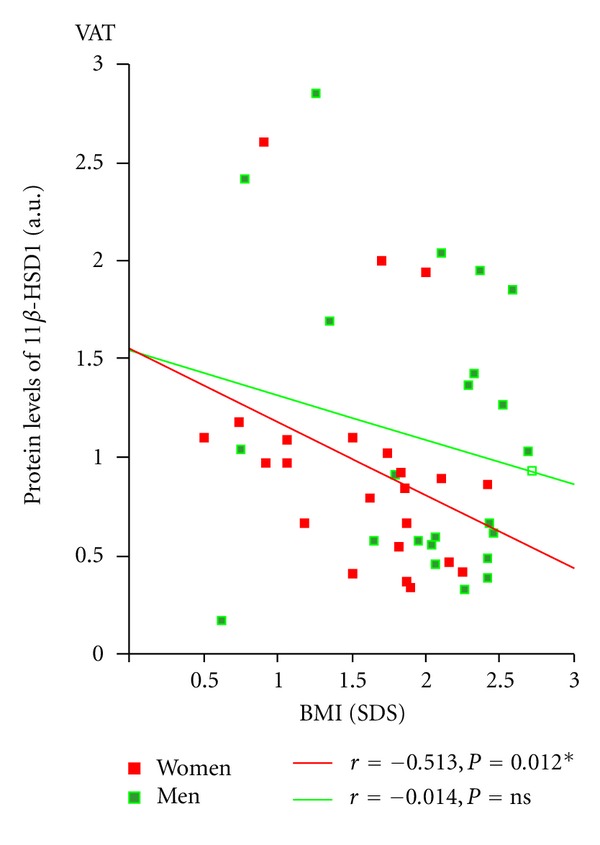
Relationship between protein levels of 11*β*-HSD1 in visceral adipose tissue and body mass index (SDS) in the whole group separated by gender.

**Figure 4:**
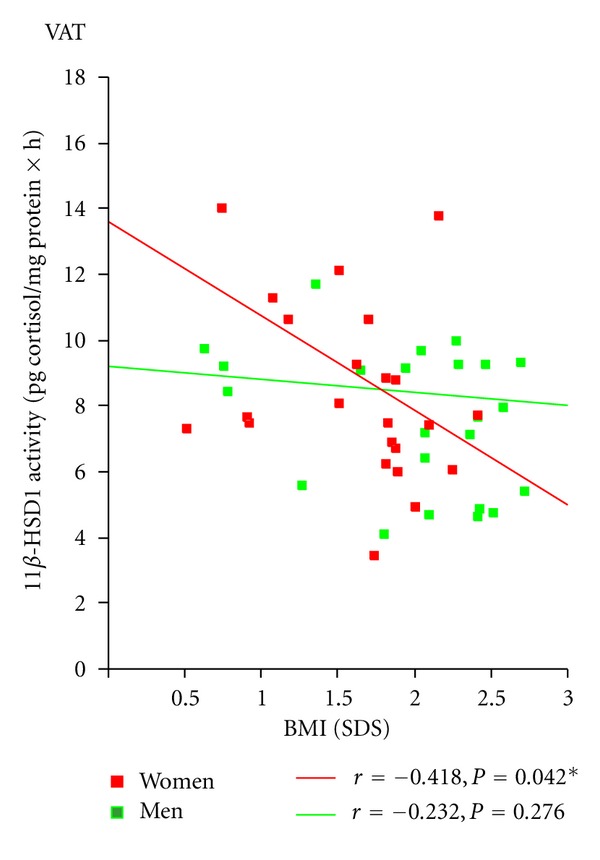
Relationship between the enzymatic activity of 11*β*-HSD1 in visceral adipose tissue and the body mass index (SDS) in whole group separated by gender.

Combining all subjects (obese and nonobese), we found a significant negative correlation between 11*β*-HSD1 enzyme activity and BMI (SDS) (*r* = − 0.290; *P* = 0.046) (data not shown). We next separated the sample by gender and found that the significant correlation was held only in women (*r* = − 0.418; *P* = 0.042; Figure 4). Similarly, an inverse correlation was found between the enzyme protein content of VAT and BMI (SDS) in women but not in men (*r* = − 0.513; *P* = 0.012; Figure 3). These correlations were not observed in SAT. 

We also analyzed whether any difference of 11*β*-HSD1 enzyme expression or activity was present between obese (M + W) subjects with a worst metabolic profile using HOMA-IR and triglycerides tertiles. Comparing the higher to the lower tertiles of these parameters, we did not find differences within the obese subjects (data not shown).

## 4. Discussion

In the present study, we analyzed possible changes in gene expression, activity, and protein content of 11 *β*-hydroxysteroid dehydrogenase type 1, involved in the metabolism of cortisol, in subcutaneous (SAT) and visceral adipose tissue (VAT) of obese adults of both genders undergoing bariatric surgery. In addition we compared the results with those obtained in a nonobese adult population. We also determined the concentrations of serum glucose, insulin, total cholesterol, and triglycerides in both the obese and in the nonobese groups.

In the present investigation, we selected a gender homogeneous group of obese subjects, and as expected, significant differences in weight and BMI for both men and women in comparison with their respective controls were observed.

 Gene expression of 11*β*-HSD1 showed no differences when comparing the obese group versus nonobese group, consistent with that reported by Cooper and Stewart [17]. Mariniello et al., however, found higher expression of 11*β*-HSD1 in VAT of obese compared with controls perhaps due to a smaller sample size [15]. The authors propose that BMI may be a factor that might influence the results. The average BMI in the report by Mariniello et al. [15] was 44 kg/m^2^ and in the report by Cooper and Stewart [17] was 33 kg/m^2^, the latter being closer to our study group (35 kg/m^2^), where similar results were found. Although the gene expression of 11*β*-HSD1 did not differ when comparing obese and controls, we found a lower expression of 11*β*-HSD1 in the VAT of women. 

To go one step further in finding the causes of the altered metabolism of cortisol in adipose tissue, we next proceeded to determine the protein content and enzyme activity of 11*β*-HSD1 in this tissue. According to the results obtained in the present study and those reported by Kannisto and Mariniello, the 34 kDa molecular form of 11*β*-HSD1 apparently is not playing an important role in the adipose tissue, leading us to focus on the 50 kDa form. The presence of this form was characteristic of this type of tissue and had a higher relative abundance compared to the 34 kDa isoform. In the obese subjects, we found a lower protein content of the 50 kDa form in VAT of obese compared to nonobese women, which was not observed neither in SAT from women nor in VAT or SAT of men, and these protein levels were negatively associated with BMI only in women. Although evidence remains unknown on the biological activity of this form, this paper set a precedent for further studies of this 50 KDa form.

Regarding the enzymatic activity of 11*β*-HSD1, the results were similar to those found with the protein content. The 11*β*-HSD1 enzyme activity according to gender was reduced in obese in VAT compared to nonobese women. These results were not found in men. This reduced activity of 11*β*-HSD1 in VAT found in women was negatively associated with BMI, which also was not observed in men, suggesting that the metabolism of cortisol through 11*β*-HSD1 enzyme is regulated in VAT with a sexual dimorphism. 

There are few reports that have measured the enzyme activity of 11*β*-HSD1 in adipose tissue. Although it has been found that both the activity and expression of the enzyme in SAT generally maintain a positive association with BMI [17, 18], the results in VAT are controversial. In one case, there was no difference in activity between obese and lean subjects suggesting a possible effect of the concentration of NADPH cofactor in the modulation of enzyme activity [19], while in the other study a direct relationship between visceral 11*β*-HSD1 activity with BMI and visceral fat mass was reported [20]. A possible contribution of macrophages in the modulation of the enzyme activity, although to a lesser extent, has been proposed [21].

In mice available reports show a clear association between the effect of the anatomical distribution of adipose tissue and the development of typical features of the metabolic syndrome, in which the visceral adipose tissue plays a key role compared to SAT. However, the results in terms of gene expression and enzyme activity of 11*β*-HSD1 in studies in visceral and subcutaneous adipose tissue in humans have been controversial and have not confirmed the observations in murine models. Whilst some studies have found differences in either expression and/or activity of the enzyme 11*β*-HSD1 in VAT of obese compared to controls [15] or between VAT and SAT of obese subjects [11, 22], others have found no differences between obese and nonobese women [11].

Our results suggest the existence of a possible modulator mechanism of the enzymatic activity of 11*β*-HSD1 in adipose tissue of women that is different from men.

This decrease in enzyme activity is the product of the lower expression and protein content of 11*β*-HSD1 in VAT of women. These findings could lead to a decrease in portal cortisol concentrations protecting the organism from the deleterious effect of visceral obesity. However, the total amount of fat mass of obese individuals is probably high enough that this compensatory effect might be attenuated. These findings must be confirmed in future studies including a higher number of subjects. Recently, the contribution of hepatic and adipose-derived cortisol in the regulation of the HPA-adrenal axis has been explored in a model of transgenic mice with a liver-specific deletion of 11*β*-HSD1 [23]. The authors showed that these LKO mice were able to regenerate cortisol from cortisone to 40% of control and circulating corticosterone was unaltered, but adrenal size was increased, indicative of chronic HPA stimulation. They conclude that liver-specific deletion of 11*β*-HSD1 reduces corticosterone regeneration and may be important for setting aspects of HPA axis tone, without impacting upon urinary steroid metabolite profile, highlighting the contribution of crosstalk between glucocorticoids target tissues in determining the metabolic phenotype.

In summary, we report a lower expression and enzyme activity of 11*β*-HSD1 in VAT of obese compared to nonobese women, which would result in a reduced local production of cortisol.

## Figures and Tables

**Table 1:** Anthropometric and biochemical characteristics of study subjects.

		Men		Women	
		Obese (16)	Nonobese (7)	Obese (16)	Nonobese (8)
Age	(years)	37.8 ± 2.5	49.7 ± 3.9	42.3 ± 3.2	39.4 ± 2.6
Weight	(Kg)	108.88 ± 2.24*	83.4 ± 3.3	93.63 ± 3.7*	68.00 ± 2.25
	(SDS)	2.24 ± 0.09*	1.01 ± 0.21	1.97 ± 0.1*	0.86 ± 0.16
Height	(m)	1.74 ± 0.01	1.75 ± 0.02	1.63 ± 0.02	1.62 ± 0.02
	(SDS)	−0.31 ± 0.16	−0.24 ± 0.23	0.00 ± 0.23	−0.23 ± 0.29
BMI	(Kg/m^2^)	35.80 ± 0.66*	27.2 ± 0.9	35.05 ± 1.14*	25.97 ± 0.66
	(SDS)	2.34 ± 0.06*	1.18 ± 0.18	1.90 ± 0.06*	0.99 ± 0.11
Total cholesterol	(mg/dL)	220.2 ± 6.8	187.4 ± 14.6	215.6 ± 8.9	159.3 ± 21.9
Triglycerides	(mg/dL)	183.5 ± 12.8	217.3 ± 75.8	201.9 ± 30.5*	81.7 ± 18.3
Insulin	(*μ*U/mL)	21.5 ± 5.9*	9.7 ± 0.7	11.9 ± 1.0	8.7 ± 0.5
Glycemia	(mg/dL)	115.4 ± 11.8	100.1 ± 2.2	92.0 ± 2.1	92.2 ± 3.1
HOMA-IR		6.6 ± 2.1*	2.6 ± 0.3	2.6 ± 0.3	2.0 ± 0.2

The data are expressed as mean ± SEM. *P < 0.05 obese versus nonobese by gender.

## Abbreviations

11*β*-HSD1: 11 hydroxysteroid dehydrogenase type 1;
VAT: Visceral adipose tissue;
SAT: Subcutaneous adipose tissue.
